# Public Health and School Children

**Published:** 1910-01-22

**Authors:** 


### The Hospital

A Journal of

The Medical Sciences and Hospital Administration.

New Series. No. 153, Vol. VI. [No. 1225, Vol. XLVII.] SATURDAY, JANUARY 22, 1910

### PUBLIC HEALTH AND SCHOOL CHILDREN.

One of the most interesting and valuable reports that have ever had the honour of appearing between blue covers is the Annual Report of the Chief Medical Officer to the Board of Education which was published this week. It is a report, we venture to assert, that should be read by every medical man and every school official; parts of it, if not the whole, should be brought to the attention of every parent or guardian whose child or charge is sent to a public or board school. Dr. Newman's reports are always illuminative, always instructive; this one is doubly so, inasmuch as it chronicles the work that has been done during the past twelve months under the 1907 Act, which provides for the medical inspection of school children. That Act came into force in 1908 and now has had a fair trial, and although there are minor points in its working and its results at which a professional critic might reasonably carp, it has had, on the whole, an amount of success which is as interesting as it is satisfactory. Let it be remembered that the Act was in a measure revolutionary, that it provided for State interference, obligatory inspection, and summary reporting in definite cases—all points to which the public, fully educated up to the idea that personal liberty, whether in things hygienic or things political, is the first and finest asset of an Englishman, might have objected very strongly. The Act introduced remarkable innovations, some, indeed, of a drastic character, and focussed attention upon new and unconsidered problems of school hygiene and administration. It laid new responsibilities upon the two professions chiefly concerned—the medical and the pedagogic—and a new burden upon the local authorities. It was likely to create new questions of unusual difficulty and open the door to abuse. It has done neither of these. One of our daily papers that looked with suspicion upon the Act has honestly confessed that its success has been remarkable, and that the danger which lay "in the possibility that a new watertight compartment of public administration might be created apart from the general work of the public health authorities" has not been realised.

On the contrary, the Act has achieved much that the public and the profession should be thankful for, as the reading of Dr. Newman's report will show. During the twelve months in which it has been operative it has slowly but surely won the respect and approval of parents. At first the medical inspection of school children was an innovation which was distinctly unpopular. To some extent the manner in which the medical officers handled a newly-created situation accounted for this unpopularity, but the main cause lay in the fact that it was an innovation, and as such resented by a public that is inherently conservative in matters of hygiene. Now, however, parents show far less disinclination to act upon the recommendations given by inspectors. The arrangements made with the metropolitan hospitals and the realisation, however late in the day, that medical inspection, to be honestly of service to the community, must be aided by treatment, have helped to attract the public attention to the Act. At the same time, it is only fair to point out that in many communities, notwithstanding the success that the Act has had in others, its provisions are not yet fully understood, its advantages not yet fully grasped by parents and teachers. There is need for much enlightenment, and we would suggest that the education authorities print a brief précis of the Act and append to it an equally brief epitome of Dr. Newman's report, showing in popular, easily understood language, what has been achieved during the year that the Act has been in operation, and that such précis be distributed gratis to parents throughout the country. There is some reason to fear that, however well the daily press may point out the moral of this the latest blue book, its national significance will be overlooked at this time of party stress, and that within a month its importance will have been forgotten.

We purpose dealing with the report in detail on another occasion. For the present it is sufficient to emphasise its concluding words that, while broad and simple foundations have been laid, to build upon them, wisely and patiently, is the work of the future. "The question of the physical condition of the people is one of the most pressing and insistent national problems," as Dr. Newman re-

[illegible]ll of
[illegible] must be marks, and it is certain that the medical inspection of school children is helping to improve that condition. The time is far too short, the experiences of a year too few and scarcely effective enough to prove these premises, but it is safe to say that the work which is daily being carried on by our medical inspectors of schools is yielding a substantial result—a result for which the nation, no less than each separate community, should be grateful. Everyone connected with that inspection, whether directly at the schools or secondarily at the hospitals, will agree with this, and not the least important part of the success has been the knowledge gained by such systematic inspection and consideration of our juvenile population. For these reasons we commend the report to the attention of every medical man.

